# Supplementary material for: Antibacterial Effect of Acoustic Cavitation Promoted by Mesoporous Silicon Nanoparticles
Source: Int J Mol Sci. 2023 Jan 5;24(2):1065. doi: 10.3390/ijms24021065 (PMC9866259; doi:10.3390/ijms24021065)
Supplement: Supplementary file 1 [file ijms-24-01065-s001.zip › ijms-2108461-supplementary.pdf]

Supplementary information

Supplementary Materials to the article:

Antibacterial Effect of Acoustic Cavitation Promoted by Mesoporous Silicon Nanoparticles

Andrey Sviridov [1], Svetlana Mazina [2,3,4], Anna Ostapenko [1], Alexander Nikolaev [5] and Victor Timoshenko [1,6,*]

1 Faculty of Physics, Lomonosov Moscow State University, Leninskie Gory 1-2, 119991 Moscow, Russia
2 Research and Technical Centre of Radiation-Chemical Safety and Hygiene, FMBA, Schukinskaya St 40, 123182 Moscow, Russia
3 Faculty of Land and Environmental Management, State University of Land Use Planning, Kazakov St. 15, 105064 Moscow, Russia
4 Faculty of Ecology, Peoples Friendship University of Russia, Miklukho-Maklaya St. 6, 123182 Moscow, Russia
5 Faculty of Chemistry, Lomonosov Moscow State University, Leninskie Gory 1-3, 119991 Moscow, Russia
6 Phys-Bio Institute, National Research Nuclear University (MEPhI), Kashirskoye Sh. 31, 115409 Moscow, Russia
* Correspondence: timoshen@physics.msu.ru

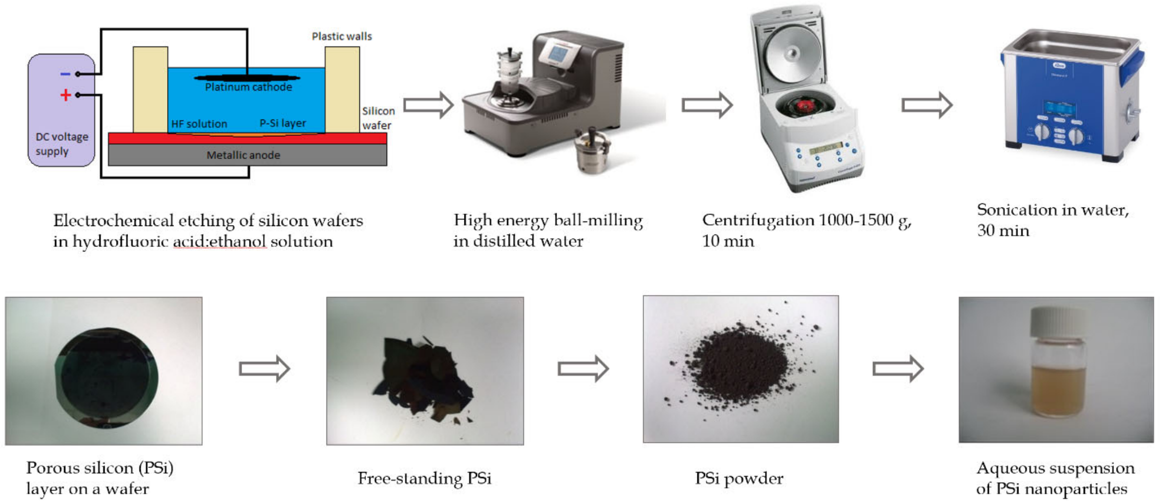

Figure S1. The synthesis cycle of m-PSi NPs. A porous silicon film is lifted off from a silicon substrate after the electrochemical etching in a HF:C_2H_5OH solution. The suspension of m-PSi NPs is obtained by the ball milling of PSi powder in water followed by centrifugation and sonication.

Supplementary information

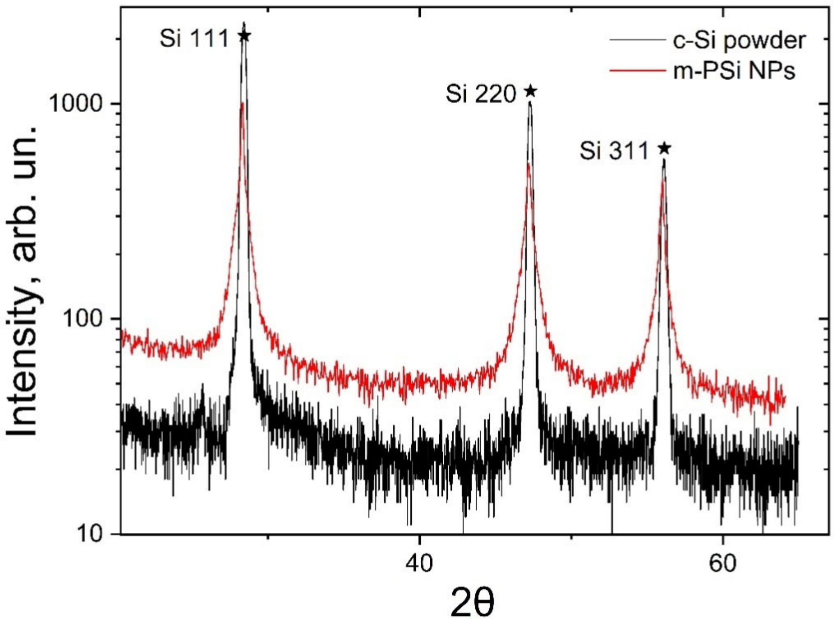

Figure S2. XRD spectra of a bulk silicon powder (**black line**) and m-PSi NPs dried from an aqueous suspension (**red line**) on a glass coverslip. Black stars indicate positions of diffraction angles corresponding to the specified Si crystal lattice planes.

Supplementary information

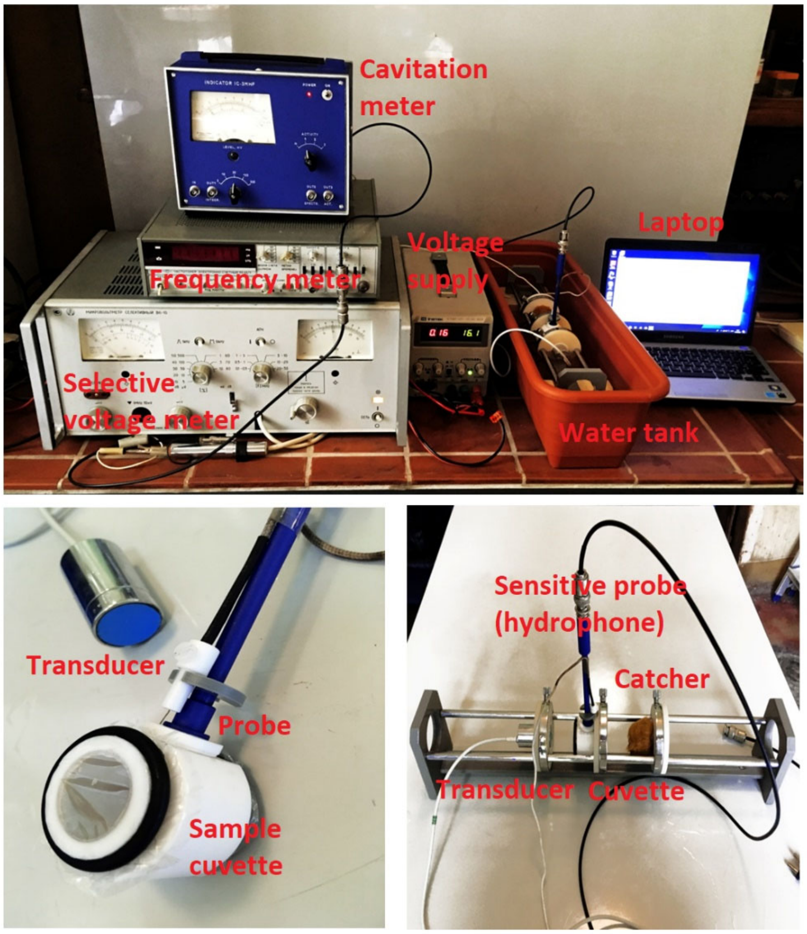

Figure S3. Photographs of the acoustic setup used for *in situ* studies of the cavitation process. Main parts of the setup are annotated in red.

Supplementary information

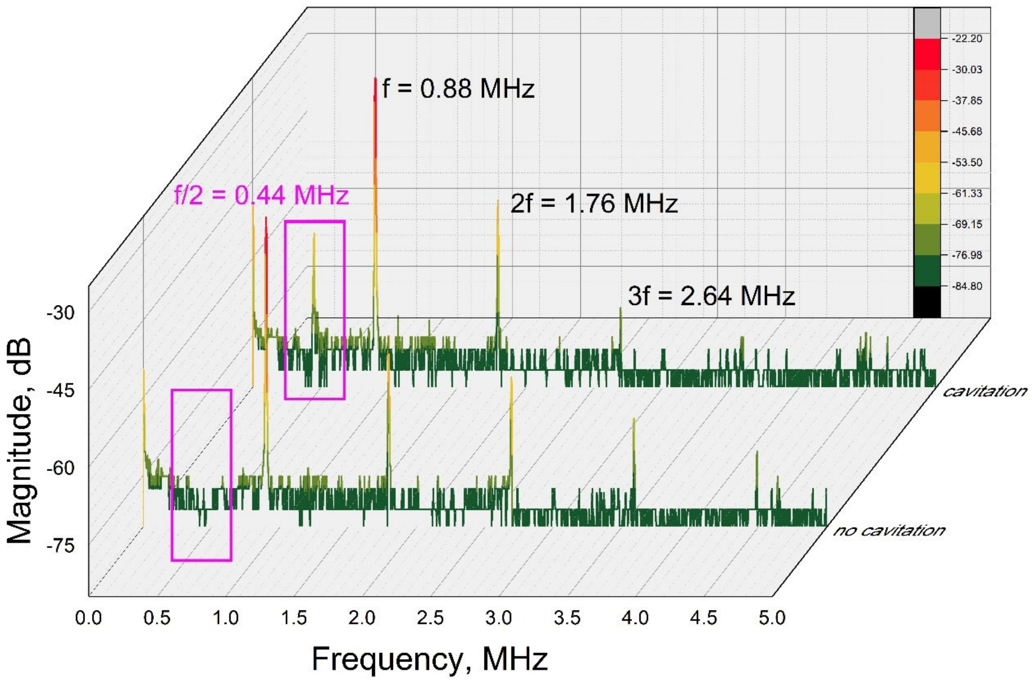

Figure S4. Typical acoustic spectra detected in a cuvette filled with m-PSi NPs where either no (in the front) or some cavitation process (in the back) takes place. The fundamental, second and third harmonics are at 0.88, 1.76 and 2.64 MHz, respectively. Frequency domains in the vicinity of subharmonic at half of the main frequency (f/2 = 0.44 MHz) framed by the magenta rectangles are indicative of the cavitation process.

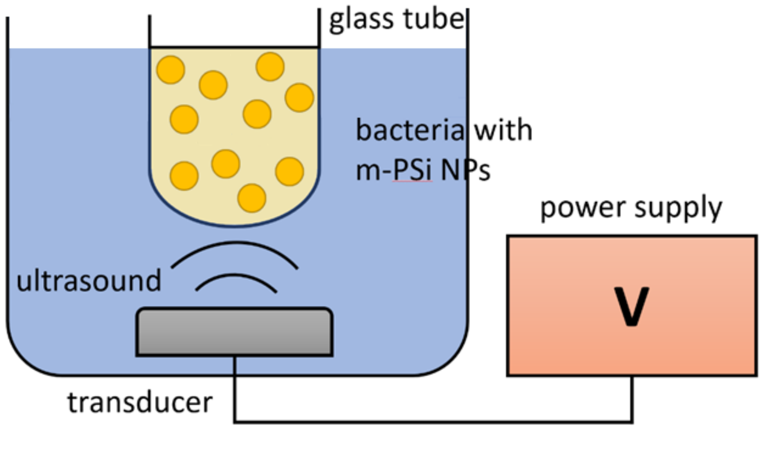

Figure S5. Schematic of the setup used for the measurement of bacteria viability under exposure to ultrasound in the presence of m-PSi NPs.

Supplementary information

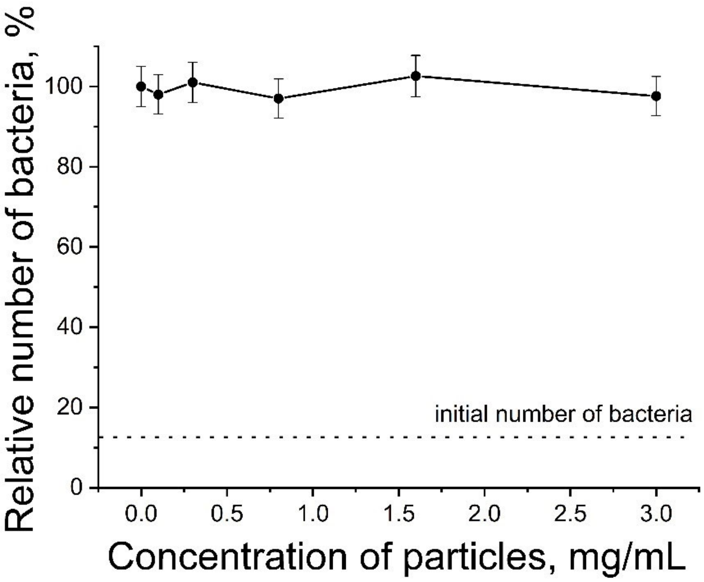

Figure S6. Viability assay of *Lactobacillus casei* after 24 h of their incubation with suspensions of m-PSi NPs added at different concentrations. The number of living bacteria is determined in a test colony relative to their number in the reference colony corresponding to zero particle concentration (addition of sterile water). The horizontal dotted line corresponds to the number of bacteria at the beginning of incubation process.
